# Automated scan quality evaluation for DDH using transfer learning: Development of a novel ensemble system

**DOI:** 10.1371/journal.pone.0317251

**Published:** 2025-03-27

**Authors:** Yeon-Kyoung Ko, Seung-Bo Lee, Si-Wook Lee

**Affiliations:** 1 Department of Brain and Cognitive Engineering, Korea University, Seoul, South Korea; 2 Department of Medical Informatics, Keimyung University School of Medicine, Daegu, South Korea; 3 Department of Orthopedic Surgery, Dongsan Medical Center, School of Medicine, Keimyung University, Daegu, South Korea; King Abdulaziz University, SAUDI ARABIA

## Abstract

**Background:**

Developmental Dysplasia of the Hip (DDH) is a relatively common hip joint disorders in infants, affecting one to three per a thousand births. If found early, it can be treated preemptively by simple non-invasive methods. But if not, then several surgical procedures may be required that can cause high economic burden. The accuracy of diagnosis using ultrasound (US) images heavily relies on locating anatomical landmarks on the image. However, there is an intra-observer/inter-observer variability in determining the exact location of the landmarks. In this study, an automated scan quality assessment system of pelvic US image by evaluating quality of five landmarks using transfer learning models was proposed.

**Methods:**

US images from 1,891 subjects were obtained at two hospitals in the Republic of Korea (henceforth Korea). Also, an ensemble system was developed using transfer learning models to automatically evaluate the scan quality by scoring five anatomical landmarks. Gradient-weighted class activation mapping was used for verifying whether models that reflect the geographical features of the images had been properly trained. Considering the applicability in the real-time environment, this study proposes an alternative sequence method (ASM) that has been discovered to have improved the lapse of scan quality assessment.

**Results:**

All the selected models achieved kappa values of 0.6 or higher, indicating substantial agreement, and the AUC score for classifying standard images based on the total score was 0.89. The activation map of the trained models properly reflected the structural features of the image. The time lapse for standard image classification was 0.35 second per image in full sequence method, and that of the three versions - ASM-1, ASM-2, ASM-3 - were 0.27, 0.22, and 0.20, respectively.

## Introduction

Developmental Dysplasia of the Hip (DDH) encompasses a spectrum of hip joint disorders, ranging from mild dysplasia to hip joint dislocation [1, 2]. It is a congenital malformation characterized by structural instability and relaxation of the hip joint capsule, affecting one to three in every thousand infants [3, 4], which varies by race and ethnicity. If found early (i.e., within the first seven weeks following the birth), it can be treated relatively simply by non-invasive methods like Pavlikarness [5], but if not, then several surgical procedures may be required, and the likelihood of success of treatment may also be decreased [6]. DDH, thus, can cause significant economic burden if not detected and treated in a timely manner [7].

Physical examination, including Barlow and Ortolani maneuvers, is less sensitive beyond neonatal period and can fail to capture mild DDH [8–10]. Hip ultrasound (US) imaging was first proposed as an alternative in the 1980s, and is widely used to diagnose DDH since then. Today, US images are typically interpreted using the Graf method [11]. The method measures an angle called “alpha” between ilium and acetabular roof, and it relies heavily on locating anatomical landmarks such as the iliac roof, labrum, triradiate cartilage, and femoral head in 2D US images [8]. However, there is an intra-/inter-observer variability as determining the exact location of the landmark in US images depends on the depth and the breadth of the practitioner’s experience [12]. Additionally, non-radiologists or clinicians unfamiliar with ultrasound may acquire inappropriate images, which may degrade the overall accuracy of computer diagnostic (CAD) systems.

An automatic assessment of the US images poses challenges due to several factors, including shadowing, image artifacts, blurred image boundaries, etc. As a result, conventional approaches, such as template-matching [13], shape-based methods [14], and feature-based methods [15, 16] face obstacles when applied to US image analysis. With the recent development of deep learning technology in artificial intelligence (AI), data-driven approaches using Convolutional Neural Network (CNN) and Recurrent Neural Network (RNN) [17] have been employed for US plane detection. Though less prevalent, methods using CNNs [7,18] and RNNs [19] have also been proposed for scan quality assessment of the hip. These techniques offer the potential for real-time evaluation of scan quality, enabling users to obtain high-quality images by identifying low-quality scans that may require repeat while the patient is still in the hospital. Additionally, automating the entire process from the acquisition of US image to the DDH diagnosis can be facilitated through the application of these methods. While the aforementioned studies have made significant advancements in automatic scan quality assessment, there still remains three primary limitations: (1) challenge in accurately identifying the quality of individual landmarks, (2) lack of consideration for prediction time, and (3) the absence of interpretation regarding the model’s predictions.

In this study, we propose an automated scan quality assessment system by evaluating the quality of five landmarks using transfer learning models. To design optimal performance in accordance with the characteristics of the US manufacturer, a transfer learning technology was used to maintain a high-performance level to cope with cases of small data volume used to train the AI. The proposed system allows users, including those unfamiliar with ultrasound, to assess the quality of individual landmarks and implements additional approaches that can improve the prediction time course, providing efficient real-time feedback. Moreover, the system enhances reliability by providing detailed interpretation of the prediction, making it a viable choice for adoption in clinical practice.

## Materials and methods

**Fig 1:**
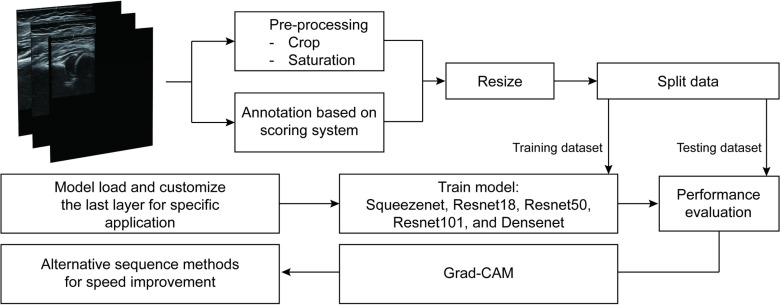
Flow chart for the entire experimental process.

The study methodology and analysis pipeline are depicted in Fig 1. US images were obtained at the hip joint, and all images were preprocessed. The dataset was divided into a model training set and a testing set for evaluation. Five pretrained models were used to assess the quality of each landmark. The performance of the artiﬁcial intelligence (AI) models and the proposed system were evaluated based on performance metrics. In the end, gradient-weighted class activation mapping (Grad-CAM) [20] was used to verify if the model accurately identifies the correct locations in the images to make predictions.

### Data acquisition

A dataset of ultrasound (US) images [21] was assembled from two different hospitals in Korea: Keimyung University Dongsan Hospital and Korea University Anam Hospital. The dataset included 49,367 US images from 1,490 subjects at Dongsan Hospital and 8,403 US images from 401 subjects at Anam Hospital. The ultrasound images were extracted from 3D US scans collected by an orthopedic specialist with ten years of experience, with an average of 30 images utilized per subject in the study. A retrospective analysis of these images was conducted with the approval of the Institutional Ethics Committee (IRB approval number DSMC 2021-04-047-013, 2022AN0125), and data access was granted on 21 April 2022, excluding information that could identify individual participants. Written consent was waived due to the retrospective nature of the study, and the study was conducted in compliance with the guidelines set out in the Helsinki Declaration. Infants were aged between 0 and 18 months (36% male, 64% female). Since DDH can be unilateral or bilateral, US images of each hip were separately collected. The specifications of the US equipment used to obtain images are as follows: HDI 5000 (Philips, Bothell, WA, USA); ACUSON SEQUOIA (Siemens medical solution, Malvern, PA, USA); iU22 (Philips Bothell, WA, USA); EPIQ 5G (Philips, Amsterdam, Netherlands); HD15 (Philips, Amsterdam, The Netherlands); RS85 (Samsung Medison, Seoul, Korea); Vivid S60 (GE Healthcare, Milwaukee, Wisconsin).

### Scan quality evaluation

**Fig 2:**
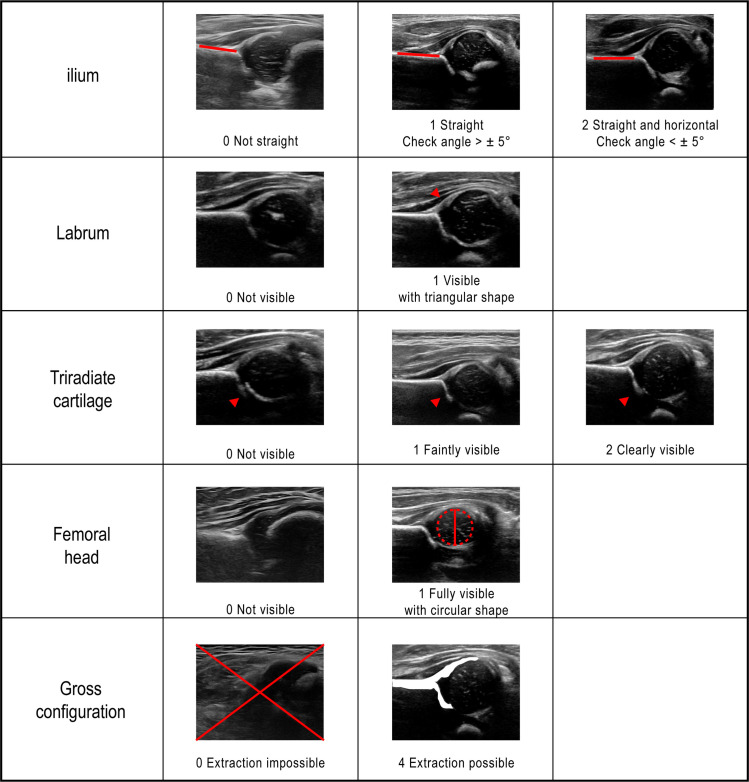
Definitions of modified scoring system for hip US images based on five landmarks - ilium (0–2), labrum (0–1), triradiate cartilage (0–2), femoral head (0–1), gross configuration (0 or 4).

A 0-10 scale scoring system was defined based on five anatomical landmarks closely associated with the Graf method: straightness of ilium; the presence of labrum; triradiate cartilage; midportion of the femoral head; and the gross configuration. In particular, the gross configuration serves as an indicator of whether the overall structure of the landmark is adequately visible, which is considered the minimum requirement for classifying an image as standard. The scoring system was designed such that images not meeting this criterion cannot be classified as standard. Fig 2 summarizes the scoring system and representative examples for each landmark.

The quality of 57,770 US images was systematically evaluated by six readers (trained for at least three months based on Fig 2 criteria) using a 0-10 scale scoring system which modified the previously published system [22] as shown in Fig 2. In cases of low inter-rater agreement, an orthopedic specialist confirmed the final quality assessment. A standard image was defined as a case wherein the total score, which is the sum of the scores of the five landmarks, reached a value of eight or higher.

### Preprocessing

The preprocessing stage of image analysis pipeline involved two main steps: cropping and saturation adjustment. In the former, the images were cropped to exclude irrelevant areas, focusing only on the region of interest (ROI) and reducing the data size processed by the network. In cases where the landmark boundary was unclear due to the black and white nature of the US images, saturation contrast enhancement techniques were applied to improve visibility. The examples of the processed image utilized in the study can be found in the S1 Fig. The resulting images served as inputs to CNNs which were pre-trained with ImageNet dataset [23].

### Transfer learning models for scan quality prediction

Instead of developing application-specific CNNs from scratch, transfer learning offers the advantage of leveraging pretrained networks with high capabilities. The fundamental precondition of transfer learning is to train a large-scale model with a diverse and extensive dataset, which then serves as a template for specific applications. Initial layers will acquire generic features (e.g., color), while the following layers will be fine-tuned to address the specific application. This approach has demonstrated its efficacy across various domains, as evidenced by the existing literature [24].

As mentioned earlier, five pretrained deep learning CNN models were employed to evaluate scan quality of hip joint US images – SqueezeNet [25], ResNet(18, 50, and 101) [26], and DenseNet-201 [27]. SqueezeNet is a lightweight CNN architecture aimed at reducing model size and computational complexity by using 1x1 filters and efficient fire modules. ResNet tackles the problem of training very deep networks by incorporating skip connections, enabling information to flow directly across layers. DenseNet employs dense connectivity, connecting each layer to every other layer. This encourages feature reuse and facilitates information propagation, resulting in improved gradient flow and stronger feature extraction capabilities. Each model shows distinct characteristics such as varying input size, network width, and the number of layers (i.e., depth). All models were pretrained using the widely adopted ImageNet dataset, with all layers except the final ones frozen to preserve the feature representations learned during pretraining. The final layers were subsequently retrained to adapt the model to the specific task of ultrasound scan quality assessment, allowing the network to leverage the generalizable features from ImageNet while fine-tuning the task-specific layers. The hyperparameters of the models were optimized using grid search, and training was terminated if there was no improvement in loss for more than five consecutive epochs. Additional details regarding the network architecture, training process, and optimization strategies have been included in the S1-S2 Tables and S2 Fig.

To ensure a fair separation between training and testing, a subject-wise split was employed. Specifically, images from 1,728 subjects were used for training and validation, and those from the remaining 163 subjects were used for testing (S3 Table). The models were retrained and validated on training set using tenfold cross validation. Subsequently, the testing set was evaluated using the trained CNN network. Performance assessment was conducted based on learning metrics, and the best-performing model for each landmark was selected as the final model to ensure the optimal outcome.

Finally, the Grad-CAM technique was employed to generate heat-maps from feature maps of each image in the testing dataset. This technique considers key areas of an image that the model has seen primarily in predicting process, and this yields a heat-maps visualization that confirms the region of the image that the model focuses on.

### Alternative sequence methods for speed enhancement.

In the context of clinical practice, the efficiency of assessing US images plays a crucial role in facilitating diagnostic assistance. Additional analysis was thus conducted to identify an effective approach for efficiently evaluating these images. The analysis was designed by prioritizing the prediction of landmark scores, which significantly contribute to the overall scoring results. For images that were unlikely to be considered as standard (i.e., total score of 7 or under), an evaluation of the remaining landmarks was skipped.

The analysis involved four different approaches: Sequence Method (SM), Alternative Sequence Method-1 (ASM-1), ASM-2, and ASM-3. In the SM approach, all five scores were predicted sequentially, starting from ilium. In the ASM-1 approach, the gross configuration was predicted first, followed by the remaining landmarks in order starting from ilium, only if the gross configuration was non-zero. In ASM-2, after predicting gross configuration and ilium, the remaining landmarks were predicted only if they were non-zero in both cases. ASM-3 predicted the remaining landmarks only if all three landmarks (gross configuration, ilium and triradiate cartilage) were non-zero.

By classifying images as standard or non-standard based on the total score calculated using each approach, the performance differences were examined. This evaluation aimed to determine whether the proposed approaches actually led to performance enhancement compared to the original method.

### Performance evaluation metrics

This study employed the metrics in Equations (1)-(6), so as to evaluate the performance of the models’ landmark score prediction and the performance of ensemble systems that classify standard and non-standard images based on the total score. The landmarks which are divided into three classes were evaluated by the average value of each class.

The evaluation metrics included the recall, which assessed the system’s capacity to identify standard images correctly, as well as the specificity, which evaluated the system’s ability to identify non-standard images correctly. In addition, the precision was used to determine the percentage of true positive images (i.e., standard) among all positive images that embrace false positives, while the accuracy measured the ratio of correctly identified positive and negative cases to the total number of images. Furthermore, the F1 score was used as a suitable accuracy indicator for datasets that are imbalanced and contain significantly different numbers of images in each class [28].

The evaluation process also employed the receiver operating characteristic (ROC) curve and the corresponding area under the curve (AUC) to assess the system’s performance. The ROC and AUC were useful in demonstrating the trade-offs between the false positive rate (1 - specificity) and the true positive rate (recall) that result from adjusting the threshold to classify cases as positive. That is, it shows the effect of changing the threshold to classify cases as positive. In order to assess the agreement between the AI predictions and manual readings, the descriptive statistics were computed, specifically using the kappa statistic.


$$\text{Accuracy}=\frac{TP+TN}{TP+FP+TN+FN}$$ (1)

$$\text{Recall}=\frac{TP}{TP+FN}$$ (2)

$$\text{PPV}=\frac{TP}{TP+FP}$$ (3)

$$\text{NPV}=\frac{TN}{TN+FN}$$ (4)

$$\text{SPC}=\frac{TN}{TN+FP}$$ (5)

$$\text{F}1\text{score}=2\times \frac{PPV\times Recall}{PPV+Recall}$$ (6)

## Results

**Fig 3:**
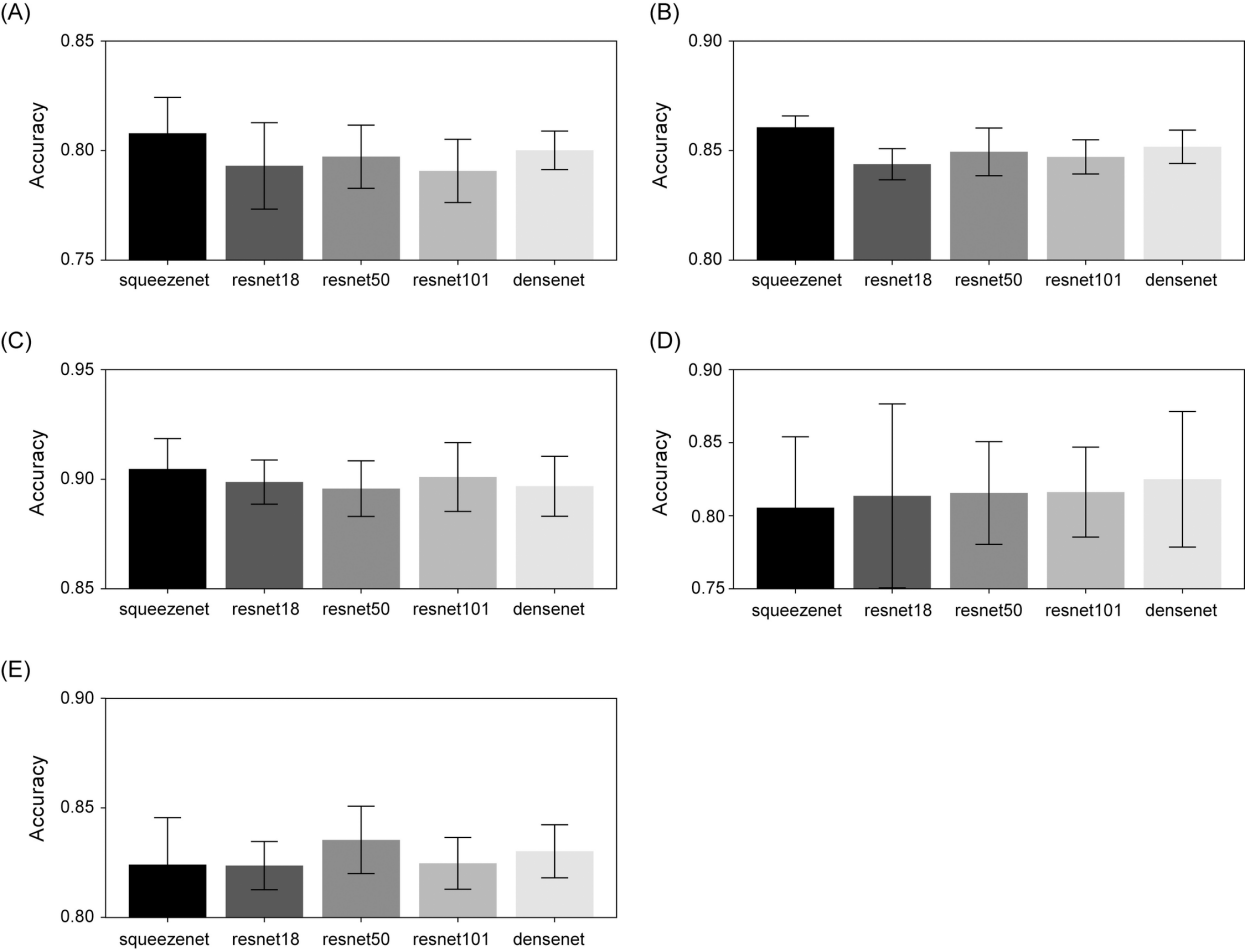
Bar graphs of mean accuracy and standard deviation of each landmark for all five pre-trained models. (A) ilium, (B) labrum, (C) triradiate cartilage, (D) femoral head, and (E) gross configuration.

The mean accuracies for all the retrained models are shown in Fig 3. The figure also gives a performance fluctuation indication of the testing set for each model trained on different folds. Compared to others, it can be seen that the performance of each fold varies greatly in predicting score of femoral head. Among the five model, the model with the highest mean accuracy was selected as the final model for predicting the score of each landmark. The SqueezeNet produced the highest mean accuracy for ilium, labrum, and triradiate cartilage. For femoral head and gross configuration, the highest mean accuracy was achieved using DenseNet and ResNet50, respectively.

**Fig 4:**
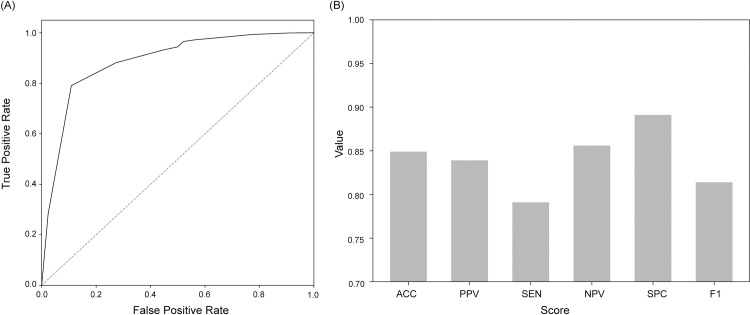
ROC Analysis and Performance Metrics of our final AI-based scan quality assessment system based on total score. (A) A ROC (Receiver Operating Characteristic) curve for the predicted total score. (B) Bar graph of performance metrics: ACC, PPV, SEN, NPV, SPC, and F1.

Detailed classification performance of the selected models is presented in Table 1. All five models showed an accuracy of 0.85 or higher, and in the case of triradiate cartilage the accuracy was 0.94. The prediction time in Table 2 means the time taken to predict the testing set for each landmark. The prediction of the femoral head required 82.20 seconds, which was relatively longer than the prediction time for other landmarks. The total score was calculated as the sum of the five predicted scores. Standard image classification performance using total score which is summation of scores of five landmarks is expressed by the ROC curves and bar graph in Fig 4. The AUC and ACC values were 0.89 and 0.85, respectively.

**Table 1:** Performance of AI based scan quality assessment models for each landmark.

Landmark	Model	No.Class	ACC	SEN	PPV	NPV	SPC	F1	Prediction time
Ilium	SqueezeNet	3	0.89	0.62	0.69	0.89	0.89	0.65	40.53
labrum	SqueezeNet	2	0.87	0.93	0.91	0.71	0.67	0.92	39.56
Triradiate cartilage	SqueezeNet	3	0.94	0.71	0.75	0.88	0.89	0.73	40.02
Femoral head	DenseNet	2	0.87	0.74	0.76	0.92	0.91	0.75	82.20
Gross configuration	ResNet50	2	0.86	0.91	0.89	0.80	0.77	0.90	50.50

**Table 2:** Performance comparison of the proposed method with previous methods.

	This study	Ref [7]
Ilium Kappa	0.67	0.66
labrum Kappa	0.61	0.33
Triradiate cartilage Kappa	0.65	0.63
Femoral head Kappa	0.66	0.66
Gross configuration Kappa	0.68	–
Total score ACC	0.85	–
Total score AUC	0.89	–

The proposed system was compared with other scan quality assessment studies (Table 2). All five models show kappa value above 0.6, which suggests substantial agreement [29]. For ilium, labrum, and triradiate cartilage, the kappa values of the proposed models were all higher than Hareendranathan et al [7] (which developed CNNs based on similar 10-point scoring system) [22] (Table 2).

**Fig 5:**
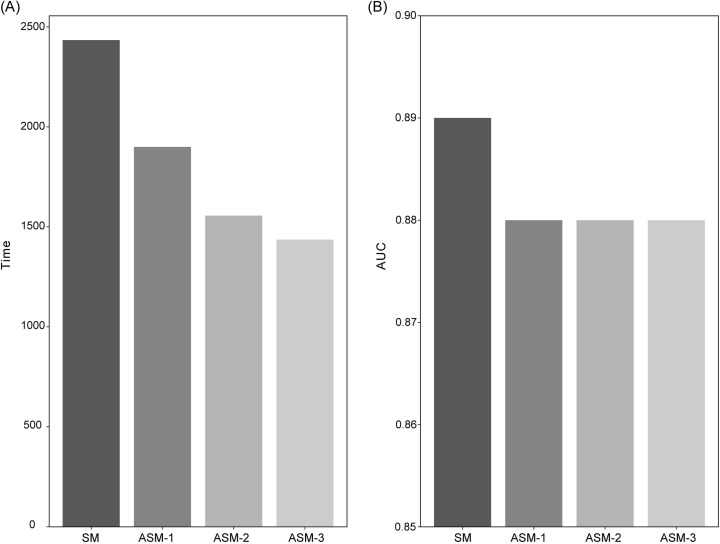
Bar graphs of secondary study results. (A) Time required for predicting, (B) AUC of each approach - SM: sequential prediction; ASM-1: gross configuration predict first, then predict sequentially; ASM-2: gross configuration, ilium predict first, then predict sequentially; ASM-3: gross configuration, ilium, triradiate cartilage predict first, then predict sequentially.

Fig 5 shows the result of additional study for alternative sequence methods for speed improvement. Compared to the SM, the lapse for predicting total score was reduced by 22%, 36%, and 41% for ASM-1, ASM-2, and ASM-3, respectively (Fig 5A). The standard image evaluation performance of the total score calculated in four methods is presented as AUC in Fig 5B. The AUCs of the three proposed methods were all 0.88. This suggests that the difference between these three AUCs and that of SM is marginal at most. The time for standard image classification per image was 0.35, 0.27, 0.22, and 0.20 seconds in SM, ASM-1, ASM-2, and ASM-3, respectively.

**Fig 6:**
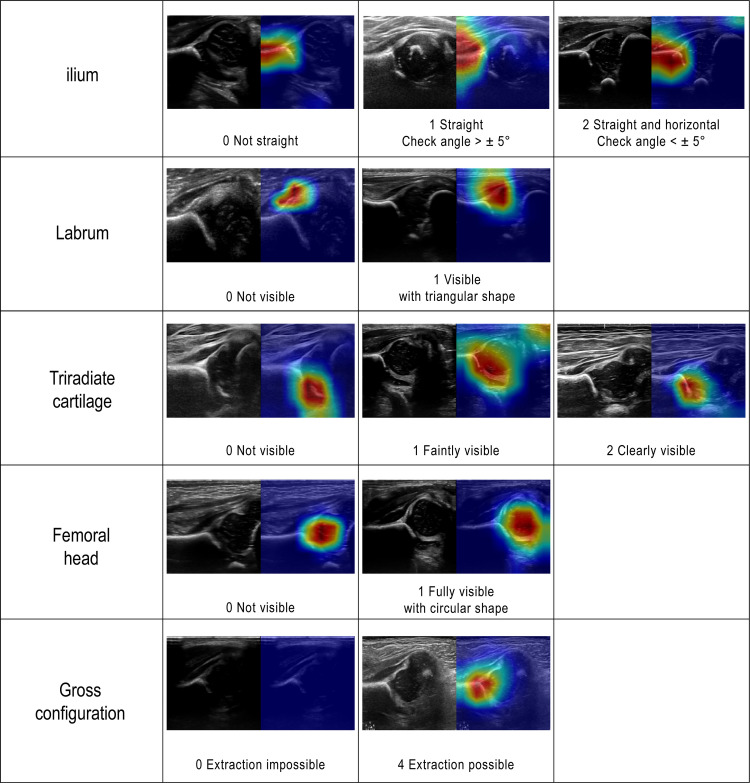
Original US image and Grad-cam result for each landmark from selected model.

This study also investigated the pre-trained models to show how they learn and evaluated the quality of landmarks in US images during testing phases. In doing so, we utilized Grad-CAM. Fig 6 shows heatmap visualization from a finally-selected model for the critical areas in US image quality assessment in each anatomical landmark. In the Grad-CAM heatmap, the red area is one that received more attention from the model. While some images, such as score 2 of ilium and score 1 of triradiate cartilage, had shown to activate regions that deviate from the relevant areas, the majority of images demonstrated that the models’ focus aligns with the expected definition of each landmark.

## Discussion

This study proposes a new ensemble system to systematically evaluate the quality of hip US scan images. The AUC of the proposed system was 0.89 and kappa values of models for all five landmarks were above 0.6, affirming better performance compared to previous studies. The proposed system was able to derive high performance with relatively little learning data by using transfer learning. The prediction time was checked to confirm applicability in the clinical practice and was significantly improved by prioritizing detailed deep learning models for standard image classification. We confirm that the proposed method increases the likelihood of clinical application by reducing the time required to evaluate all images contained in testing set by up to 41%. Furthermore, we utilized Grad-CAM to confirm that the proposed model is well focused on the areas in the image for scan quality prediction.

The proposed system is an ensemble of pre-trained models that showed the best performance for each landmark. The model structure, which showed the highest accuracy, was different by landmark, and this attributes to the difference in the size of the area in which the model focuses on to evaluate quality. Specifically, for smaller anatomical structures like ilium, labrum, and triradiate cartilage, the SqueezeNet outperformed the others (Fig 3). Squeezenet is a model developed for the purpose of reducing computation and learning speed rather than classification performance. So it shows lower classification performance than ResNet and DenseNet in general [30, 31]. However, in the problem of classification focusing on smaller areas, it seems that the SqueezeNet achieved better performance compared to other models because fewer learning parameters were more advantageous. On the other hand, for larger areas such as femoral head and gross configuration, ResNet and DenseNet, both of which have more capacity for learning, yielded the best performance of all in this study. The study proposed a system that combines optimal models which can vary depending on the size of these anatomical structures. The ensemble approach, as demonstrated by the proposed method, proves to be an advanced option when in comparison with the cases in which one relies solely on a single model as in, for example, Paserin et al [19].

We also conducted further analysis to confirm the clinical applicability of the proposed system. The analysis was designed by prioritizing landmark score predictions that contribute significantly to a 0-10 scale scoring system. There is little difference in AUC performance between original and proposed methods, but the time spent on prediction is reduced by up to 41% (Fig 5). This demonstrates that the proposed method (specifying the prediction order according to the priority of each landmark) increases the efficiency in scan quality assessment.

Finally, the study presented the Grad-CAM results of the final model for each landmark (Fig 6). The study discovered that models we developed accurately predict scores by focusing on the relevant regions associated with each landmark, despite utilizing pre-trained models trained on data from non-medical domains. The Grad-CAM visualization provides insights into the features extracted by the network for making predictions. This interpretability of the model’s output enhances the reliability of the entire framework, as it enables physicians to immediately understand the reasoning behind the quality assessment. This is pivotal in the field of medicine, as it contributes to the explainability of scan quality prediction and increases the likelihood of clinical acceptance of the proposed method.

Despite numerous benefits, there remains limitations in this study. First, inter-reader variability in ground truth can potentially undermine the achievements in this study as the assessment is processed manually. The scoring system aimed to reduce variability by simplifying image quality evaluation through landmark-based evaluation, but there was no perfect consensus about the scoring criteria, especially for the cases of images with ambiguity and mediocre quality. Second, there was an imbalance in the distribution of images across different scores for each landmark (S1 Table). The model’s performance can be influenced by the number of data available in each class, resulting in lower sensitivity and PPV as shown in Table 1. Finally, models in this study were developed based on offline learning using US images collected from two hospitals. Offline learning has a limitation that the performance of the model is limited to the data it was trained on. Thus, performance may vary depending on the specific US imaging equipment and display type, requiring retraining.

## Conclusion

This study introduced a novel AI-based system for automatically assessing US image scan quality, achieving strong performance in predicting landmark quality and classifying standard images (AUC 0.89). The system provides real-time feedback with a 0.2-second processing time per image, potentially improving the efficiency of image acquisition. By training models for five anatomical structures, we also generated Grad-CAM results, offering physicians clearer insights into the quality assessment process and enhancing transparency.

## Supporting information

S1 Fig Example of preprocessed ultrasound image. (PDF)

S2 Fig The architecture of 5 utilized models; Squeezenet (A), Resnet18 (B), Resnet50 and Resnet 101 (C), and Densenet (D). (PDF)

S1 Table Models’ detailed information. (PDF)

S2 Table Hyperparameter selection through grid-search. (PDF)

S3 Table Feature prevalence of five landmarks - ilium (0–2), labrum (0–1), triradiate cartilage (0–2), femoral head (0–1), gross configuration (0 or 4). (PDF)

## Abbreviations

DDH: Developmental dysplasia of the hip
US image: Ultrasound image
ROC curve: Receiver operating characteristic curve
AUC: Area under the curve.
