# Supplementary material for: Automated scan quality evaluation for DDH using transfer learning: Development of a novel ensemble system
Source: PLoS One. 2025 Mar 27;20(3):e0317251. doi: 10.1371/journal.pone.0317251 (PMC11949359; doi:10.1371/journal.pone.0317251)
Supplement: S1 Fig — (PDF) [file pone.0317251.s001.pdf]

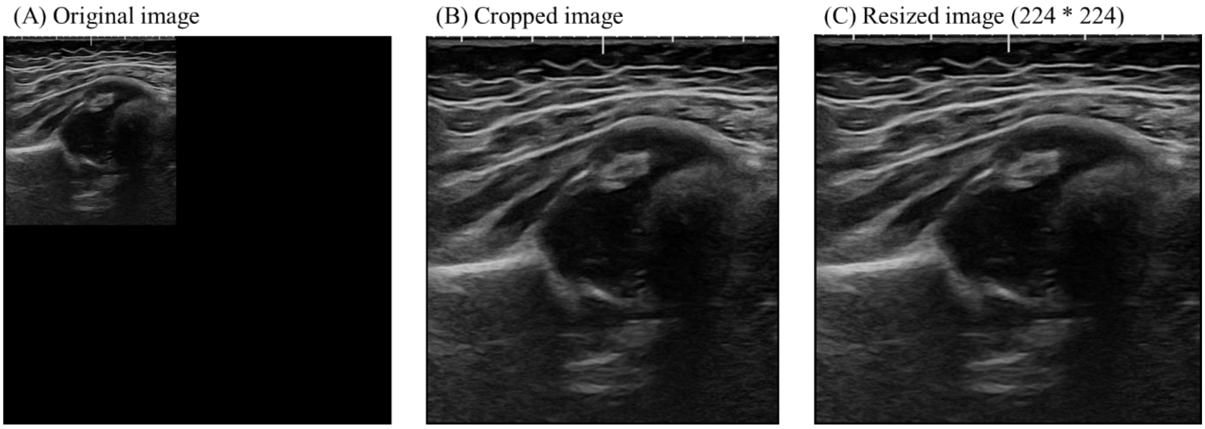

S1 Fig. Example of preprocessed ultrasound image
