# Supplementary material for: Automated scan quality evaluation for DDH using transfer learning: Development of a novel ensemble system
Source: PLoS One. 2025 Mar 27;20(3):e0317251. doi: 10.1371/journal.pone.0317251 (PMC11949359; doi:10.1371/journal.pone.0317251)
Supplement: S1 Table — (PDF) [file pone.0317251.s003.pdf]

S1 Table. Models' detailed information

Model	# layers	# params
Squeezenet	26	736,963
Resnet18	18	11,177,538
Resnet50	50	23,512,130
Resnet101	101	42,504,258
Densenet	121	7,980,906
