# Supplementary material for: Automated scan quality evaluation for DDH using transfer learning: Development of a novel ensemble system
Source: PLoS One. 2025 Mar 27;20(3):e0317251. doi: 10.1371/journal.pone.0317251 (PMC11949359; doi:10.1371/journal.pone.0317251)
Supplement: S2 Table — (PDF) [file pone.0317251.s004.pdf]

S2 Table. Hyperparameter selection through grid-search

Hyperparameter	Search space	Selected value
Optimizer	SGD, Adam	SGD
Learning rate	0.001, 0.005, 0.01	0.005
Batch size	32, 64, 128, 256	128
Loss function	Cross entropy, Focal loss	Cross entropy
