# Supplementary material for: Automated scan quality evaluation for DDH using transfer learning: Development of a novel ensemble system
Source: PLoS One. 2025 Mar 27;20(3):e0317251. doi: 10.1371/journal.pone.0317251 (PMC11949359; doi:10.1371/journal.pone.0317251)
Supplement: S3 Table — (PDF) [file pone.0317251.s005.pdf]

S3 Table. Feature prevalence of five landmarks - ilium (0–2), labrum (0–1), triradiate cartilage (0–2), femoral head (0–1), gross configuration (0 or 4)

Landmark	Training set			Testing set		
	0	1	2	0	1	2
ilium	22,892	3,647	23,592	3,750	172	3,714
Labrum	9,469	40,662	-	1,646	5,990	-
Traiated cartilage	5,832	42,735	1,564	911	6,619	106
Femoral head	32,762	17,369	-	5,747	1,889	-
Gross configuration	15,662	34,469	-	2,492	5,144	-
